# Examining the Planning Policies of Urban Villages Guided by China’s New-Type Urbanization: A Case Study of Hangzhou City

**DOI:** 10.3390/ijerph192416596

**Published:** 2022-12-10

**Authors:** Yue Wu, Yi Zhang, Zexu Han, Siyuan Zhang, Xiangyi Li

**Affiliations:** 1 Department of Architecture, Zhejiang University, Hangzhou 310058, China; 2 International Center for Architecture & Urban Development Studies, Zhejiang University, Hangzhou 310058, China; 3 China Institute for New Urbanization Studies, Zhejiang University, Hangzhou 310058, China

**Keywords:** urban village, planning policies, new-type urbanization, formality–informality

## Abstract

Planning policies have greatly influenced the development of urban villages, an informal phenomenon in which rural settlements are encircled by urban environments during China’s rapid urbanization process. “The National New-type Urbanization Plan (2014–2020)” of China initiated in 2014 provides a new perspective on planning policy research on China’ urban villages. Hangzhou, a pioneer city that adopts new-type urbanization in China and combines the characteristics of rapid urban growth, mountainous urban terrains, and a long cultural history, serves as a typical case study to compare the planning policies responding to the informality of urban villages guided by traditional and new-type urbanization. This study employed the content analysis method to analyze the evolution of Hangzhou’s planning policies of urban villages since the reform and opening up and used one-way ANOVA to analyze the differences in rental levels among the urban villages developed under the planning policies of different urbanization stages, aiming to compare the influences of planning policies guided by traditional and new-type urbanization on urban village development. The results indicate that the policies allowing some degree of informality in the new-type urbanization stage achieve a higher rental level for urban villages than the policies of the traditional urbanization stages that restrict and prevent informality. The findings of this research suggest that informality may provide advantages that formality cannot replace and provides important policy implications for rapidly urbanizing countries.

## 1. Introduction

China has experienced the largest and fastest process of urbanization in world history, with its urbanization rate increasing from 17.9% in 1978 to 53.73% in 2013 [1], and the country’s local governments and related policies have played an important leading role in this process [2]. Urban villages are at the forefront of China’s urbanization process and represent a key urban issue concerning informality in present-day China. In China’s urban-rural dual system, urban land is owned by the state, while rural land is owned by village collectives and can be requisitioned by the local government for urban development. To achieve rapid urban expansion and prevent excessive economic and social costs, local governments usually requisition only agricultural lands for urban construction while bypassing rural settlements or designating new rural settlements for village collectives [3,4,5]. Consequently, these rural settlements that previously relied on agricultural production are gradually encircled by urbanized environments, becoming “islands” in the cities [6]. Despite the fact that urban villages have provided affordable housing for a large number of migrants and to some extent mitigated the shock of rapid urbanization on cities, these settlements are usually considered an undesirable and informal form of urban development and are part of the land banking plans of local governments, which make the related planning policies replace urban villages with controllable urban spaces through demolition–rebuilding methods [7,8]. However, previous experiences have demonstrated that although these planning policies have to some extent improved the urban environments, they have also resulted in new urban issues, including social exclusion, intensified job–housing contradictions, the outflow of migrant workers, and the lack of distinctive urban cultures [9,10,11]. Therefore, there is a great need to reconsider the role of urban villages in the urbanization process to explore new planning policies that are more appropriate for the long-term development of urban villages and cities.

New-type urbanization is a new urbanization strategy proposed by the Central Committee of the Communist Party of China (CPC) since the 18th CPC National Congress aiming to change the traditional urbanization concept that prioritizes “land” over “people”, “speed” over “quality”, and “cities” over “villages”. In 2014, the Chinese government launched “the National New-type Urbanization Plan (2014–2020)”, embarking on a transformation of the urbanization model in China [12]. The plan emphasizes the reform of urbanization mechanisms and places “people’s urbanization” at its center. It also attaches importance to a thorough awareness of the link between urban and rural areas, as well as of the local natural, historical, and cultural resources, to form a new urbanization model that is compatible with local conditions. Informality, a special phenomenon produced by local people’s spontaneous practices outside the formal institutions to make up for the inefficiency of the formal urbanization mechanisms, which may have ignored certain groups of people’s benefits, is considered an important concern in practicing China’s new-type urbanization [13]. Therefore, urban villages, a typical form of informality in China, are becoming a focus of new-type urbanization, which also provides a new cognitive perspective on the role of urban villages. It is against this background that the article examines the various planning policies responding to the informality of urban villages in Hangzhou city.

**Figure 1:**
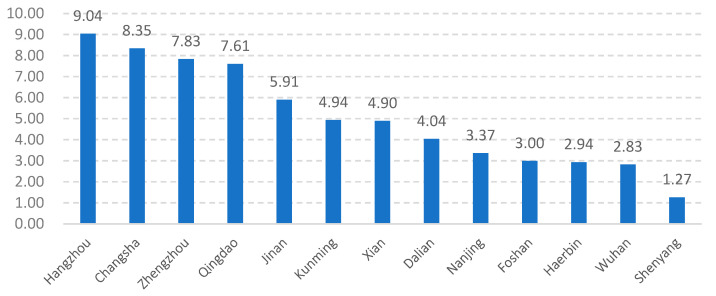
Growth rate of population of Chinese megacities from 1982 to 2020 (megacities are cities with a population of 5 million to 10 million).

Hangzhou is the capital city of Zhejiang Province, a pioneer area in practicing new-type urbanization in China. Since President Xi Jinping proposed “taking the road of new-type urbanization” at the provincial working conference in 2006, Zhejiang Province has taken the lead in China in exploring innovative reform strategies for urbanization mechanisms. With years of practice, Zhejiang Province has become one of the provinces with the greatest level and quality of urbanization in China [14]. As the capital city of Zhejiang Province, Hangzhou has also explored a wide range of cutting-edge strategies to reform the urbanization mechanisms. Additionally, Hangzhou is one of the fastest growing megacities in China (Figure 1). The urban built-up area expanded from 102 km^2^ in the 1980s to 666.18 km^2^ in 2020, during which approximately 400 urban villages proliferated in Hangzhou’s urban district, creating great challenges for urban development. Meanwhile, Hangzhou exhibits a unique topography where hills and plains coexist. The plain areas are well suited for extensive land development, and the urban villages in plain areas typically complete the rapid process of land expropriation and housing demolition and are replaced with formal urban spaces under uniform planning in the traditional urbanization stage. The hilly areas are not suitable for large-scale urban expansion due to relatively high costs, and the local government usually bypasses the urban villages in hilly areas in land development. Consequently, compared with the urban villages in plain areas, the urban villages in hilly areas in Hangzhou preserve the informality produced by local villagers’ spontaneous practices and implement a differentiated urbanization strategy with the planning policies in the new-type urbanization stage. Therefore, as a pioneer city in practicing new-type urbanization combining the characteristics of rapid urbanization, a characteristic geographical environment, and a long historical culture, Hangzhou serves as a typical case study to compare the planning policies responding to the informality of urban villages guided by traditional and new-type urbanization.

This research takes Hangzhou as a case study and aims to compare the influences of planning policies for urban villages guided by traditional and new-type urbanization on urban village development. This article is not intended to provide a paradigm model for Chinese urban villages. Instead, it seeks to comprehend the variations and impacts of local government’s cognitions of urban villages and their role in the urbanization process.

## 2. Literature Review

New-type urbanization provides a new perspective on the cognition of urban villages and serves as an important theoretical basis for this research. Moreover, informality is a research field explaining special phenomena outside the control of formal institutions in the urbanization process of developing countries and provides a theoretical background for the issues of Chinese urban villages. The analytical framework of this research is based on the theory of new-type urbanization and informality to study the planning policies and development of urban villages guided by new-type urbanization.

### 2.1. Transformation of the Cognition of Urban Villages Guided by New-Type Urbanization

New-type urbanization is a new concept of urbanization developed under the peculiar development circumstances of China. Compared with the traditional urbanization model, new-type urbanization does not differ significantly in terms of the concept of population agglomeration, growth of non-agricultural industries, and expansions of urban spaces, but it differs in terms of the methods for realizing such processes [15]. Compared with traditional urbanization, new-type urbanization exhibits at least the following three aspects of transformation. First, guided by new-type urbanization, the focus of urbanization changes from the expansion of urban physical form to the comprehensive development of “people” [16]. During the traditional urbanization process, many local governments relied excessively on land finance and considered urban expansion and economic growth the only standards to measure the level of urbanization, which led to issues, such as the loss of high-quality arable land, the growing phenomenon of “ghost cities”, the urban heat island effect, and global warming, hindering further steps of urbanization [17]. New-type urbanization emphasizes people’s urbanization, which prioritizes social and economic development, as well as complex issues related to people’s overall well-being. It also advocates for higher quality urbanization through institutional innovation in terms of the household registration system, land system, social security system, etc. [18]. Second, guided by new-type urbanization, urbanization emphasizes equal and complementary development between urban and rural areas as opposed to prioritizing growth in urban areas [19,20]. In the traditional urbanization process, which gave priority to urban development, rural areas made a substantial sacrifice for urban development with cheap land, labor, and other factors [21,22], resulting in “rural diseases”, such as industrial hollowing out, landscape fragmentation, and lack of local culture [23]. New-type urbanization requires ending the mindset of the dual urban-rural relationship and the corresponding policies [24] and exploring the strategies for differentiated and coordinated urban-rural development according to their respective development rules and local cultures [25]. Third, guided by new-type urbanization, the governments’ role changes from being completely dominant with comprehensive control to becoming a coordinator with limited control [26]. The traditional urbanization process in China is a planned process promoted by the government [27]. In this process, the civilization of farmers may become a completely institutionalized process of “being civilized”, which not only brings the dilemma of insufficient urban adaptability to these new citizens but also readily generates new urban-rural conflicts and social issues [28]. Guided by new-type urbanization, the promotion mode of urbanization shifts from “government-dominated” to “government-coordinated and multi-actor participation” to ensure that local villagers and village collectives actively participate in the urbanization process and to give better play to the market in resource allocation, that is, to combine bottom-up factors with top-down factors [29] to exert their advantages. In summary, compared with traditional urbanization, new-type urbanization shifts its emphasis from “land” to “people” and their development rights, from prioritizing urban development to supporting complementary and coordinated urban-rural development, and from being “dominated” by the government to being “coordinated” by the government, all in an effort to ensure higher quality urbanization.

The shift of the urbanization strategy also provides a new perspective for understanding the urban village and its role during the urbanization process. First, guided by new-type urbanization, the cognitive perspective of urban villages shifts from focusing on the physical form to focusing on “people”. During the traditional urbanization process, urban villages were mostly considered physical spaces that would eventually be replaced by urban environments, while the development rights of “people” were ignored. Guided by new-type urbanization, the development and planning practices for urban villages focus more on the rights of “people” for long-term development rather than simply providing one-time compensation for demolition and spatial reconstruction. Second, guided by new-type urbanization, the cognitive perspective of urban villages shifts from static to dynamic. During the traditional urbanization process dominated by the priority of urban development, urban villages were primarily seen as the land being prepared for future urban development. Guided by new-type urbanization in which urban and rural areas are regarded as equal and complementary, village-style local cultures with authenticity are more valued and recognized as an important aspect of cultural diversity in cities [30,31]. Therefore, the development of urban villages is no longer a static process in which urban villages wait to be demolished and are used for land banking but a dynamic process in which urban villages, as carriers of characteristic cultures, co-prosper with urban cultures. Third, guided by new-type urbanization, the cognitive perspective of urban villages shifts from passive acceptance of urbanization to active involvement in urbanization. During the traditional urbanization process, local governments control the whole process of land expropriation, land banking, and redevelopment, while the local villagers and tenants play a passive role in this process. Guided by new-type urbanization, local governments are coordinators creating enabling environments for the development of urban villages to stimulate the local residents’ initiative in the planning practices of urban villages and to give better play to the socioeconomic value of urban villages. In summary, guided by new-type urbanization, the cognitive perspective of urban villages shifts from focusing on the physical form to focusing on “people”, from being static to being dynamic, and from passively accepting urbanization to actively participating in urbanization.

### 2.2. Informality of Urban Villages

Informality refers to a unique phenomenon produced by spontaneous practices outside the control of formal institutions and planning control power. It is thought to be a common phenomenon during the urbanization process of many developing countries [32,33,34,35,36], appearing in the urban-rural interface with intense urban-rural interactions in terms of economic, social, and environmental aspects [37,38] under governments’ special governance logic [33,37,39]. Informality has previously been studied from a static perspective, with an emphasis on the physical spaces or category of labor, such as the informal economy, informal labor, and informal settlement [40,41,42,43,44]. Recently, more research has overcome the dichotomy between formality and informality and has seen them as an inter-related and complementary unity [45,46]. The concept of “formality–informality” has frequently been taken as a starting point to study complex and dynamic urban issues and to explore the cooperation approaches between formal institutions and local residents’ spontaneous practices to form effective resource allocation or practice promoting socioeconomic development [47,48]. Therefore, “formality–informality” provides an appropriate analytical framework to study the development of urban villages under the guidance of new-type urbanization, where the cognitive perspective of urban villages shifts from focusing on the physical form to focusing on “people”, from being static to being dynamic, and from passively accepting urbanization to actively participating in urbanization.

The informality of urban villages is mainly derived from China’s urban-rural dual structure [49,50,51], and the relevant studies on planning policy responding to informality mostly focus on the three aspects of urban-rural dual land system, spatial planning system, and public resources management system. First, there are two different types of land tenure and property rights regimes in urban and rural areas. In the late 1980s, China’s urban land use system was transformed from uncompensated allocation to compensated use and market-based transactions [52] to promote urban construction and economic growth. Urban residents became able to pay to obtain legally transferable residential land use rights. Village homestead, however, is allocated to local villagers free of charge for housing construction as welfare [53,54] and is non-transferable and non-mortgageable [55,56]. Due to these institutional barriers, urban villages cannot benefit from urbanization through formal or legal means of transactions, while the land value surrounding urban villages has greatly improved, which to some extent hinders the development of urban villages. Currently, local governments are attempting to convert collectively owned land into state ownership through requisition for further urban development to release the land value. However, a large number of these projects have resulted in long project cycles or project termination due to severe conflicts of interest among different stakeholders and continuously escalating compensation expenses [57,58]. Therefore, it is necessary to explore new policies for the homestead of urban villages to improve the land value and realize a more rational benefit allocation. Second, the spatial planning system in China has exhibited a clear urban-rural division [59]. The current planning system in China is a structural system oriented toward economic development and urban growth aiming to meet the urban development needs in the period of large-scale industrialization and rapid urbanization, while rural planning is less studied and mostly focuses on “transplanting urban consciousness” [20]. For urban villages, a unique type of rural settlement in urban areas, there is no suitable planning reference in the current planning code [60]. Therefore, urban villages are usually unplanned or roughly planned and exhibit problems of low-efficiency land use. Currently, demolishing urban villages and constructing medium- and high-rise apartments is the main spatial planning method to improve land use efficiency. Nevertheless, these practices have destroyed the unique village textures and have been considered to damage the local cultures and ecological integrity [61,62]. Accordingly, it is necessary to explore new policies concerning spatial planning to improve land use efficiency, as well as preserve the characteristic village cultures. Third, under the dual public resources management system for urban and rural areas, urban villages have not been provided with enough public services or financial support by local governments [4]. Moreover, driven by a strong interest in the rental market, local villagers, usually with weak legal and civic consciousness, tend to occupy public spaces for illegal construction [60]. Therefore, urban villages usually exhibit the characteristics of insufficient infrastructure, poor public environment, and serious fire safety issues, hindering the development of urban villages and their surrounding areas. Currently, upgrading is believed to be a suitable approach for improving the infrastructures and living conditions of urban villages. However, there is little research on how to better exert the role of village collectives to create a better public environment suited to the living demands of both local villagers and tenants, and to prevent the waste of public resources because of demand mismatch and a lack of civic consciousness [24,63]. The above research shows that the planning practices for urban villages in the past were usually guided by traditional urbanization concepts, such as paying more attention to “land” than to “people”, paying more attention to “cities” than to “villages”, and being “completely dominated by governments” with the main target of completely replacing the informality of urban villages with formal urban spaces. These planning practices may not suit the long-term development of urban villages. To better utilize informality and overcome the institutional barriers of the urban-rural dual system, including the dual land system, spatial planning system, and public resources management system, it is imperative that innovative, incremental, and adaptive policy design be implemented for urban villages, supporting the long-term development of urban villages.

In summary, guided by new-type urbanization, the cognitive perspective of urban villages shifts from focusing on the physical form to focusing on “people”, from being static to being dynamic, and from passively accepting urbanization to actively participating in it. Moreover, as a theoretical tool for analyzing the dynamic interaction between formal institutions and local residents’ spontaneous practices, “formality–informality” serves as a suitable analytical framework to study the planning practices of urban villages guided by new-type urbanization. Furthermore, the existing research usually considers urban villages to be transitional communities developed by informal survival strategies that will eventually be replaced by formal urban spaces. Less research focuses on the long-term and dynamic interactions between formal institutions and local residents’ spontaneous practices to break through the institutional barriers of the urban-rural dual land system, spatial planning system, and public resources management system and to realize incremental, innovative, and adaptive policy design and practices. Therefore, this article focuses on planning policies for urban villages that reflect local governments’ perceptions of the informality of urban villages and establishes a policy analytical framework based on the homestead, spatial planning, and upgrading, the three main aspects of planning policies responding to the informality of urban villages, to compare the planning policies guided by traditional urbanization and new-type urbanization, as well as their influences on urban village development.

## 3. Materials and Methods

### 3.1. Study Area

**Figure 2:**
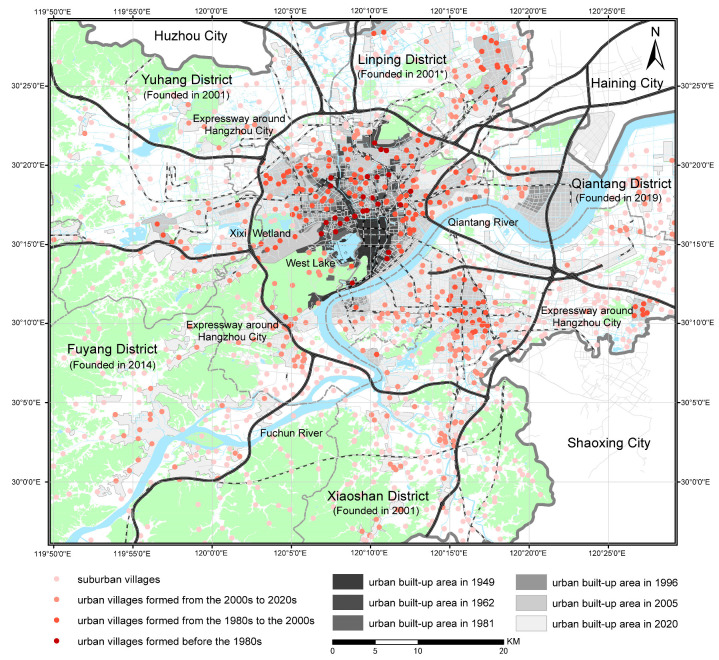
Spatial distribution of urban villages in Hangzhou urban district. * The territory of Linping District was formerly a part of Yuhang District, which was founded in 2001 and later split into the districts of Yuhang and Linping for administrative district adjustment.

**Figure 3:**
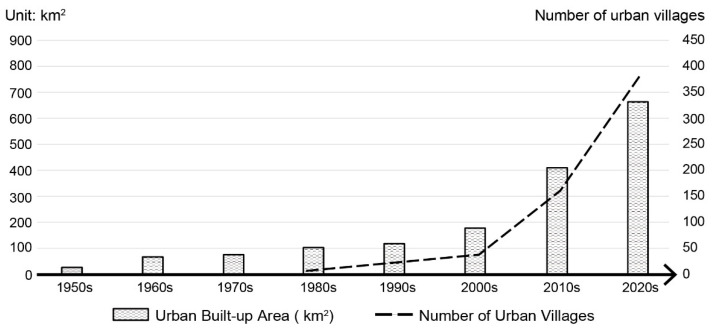
Built-up area and number of urban villages in Hangzhou urban district form the 1950s to the 2020s.

This study takes Hangzhou, the capital city of Zhejiang Province, as a case study. Figure 2 and Figure 3 show the spatial distribution of urban villages and the urbanization process of Hangzhou. Since the 1980s, Hangzhou has accelerated its urbanization process, and the built-up area in the Hangzhou urban district grew from 102 km^2^ in 1980 to 666.18 km^2^ in 2020 [64]. During that time, approximately 400 villages were gradually surrounded by urban environments and became urban villages. These urban villages were located in both plain and hilly areas and developed under different planning policies. Moreover, Hangzhou is still undergoing rapid urbanization, and over 1000 suburban villages located outside of the built-up area are expected to be increasingly integrated into the city. Therefore, Hangzhou will continue to be an experimental area in new-type urbanization and planning for urban villages, providing references for other rapidly urbanizing areas.

### 3.2. Methods

**Figure 4:**
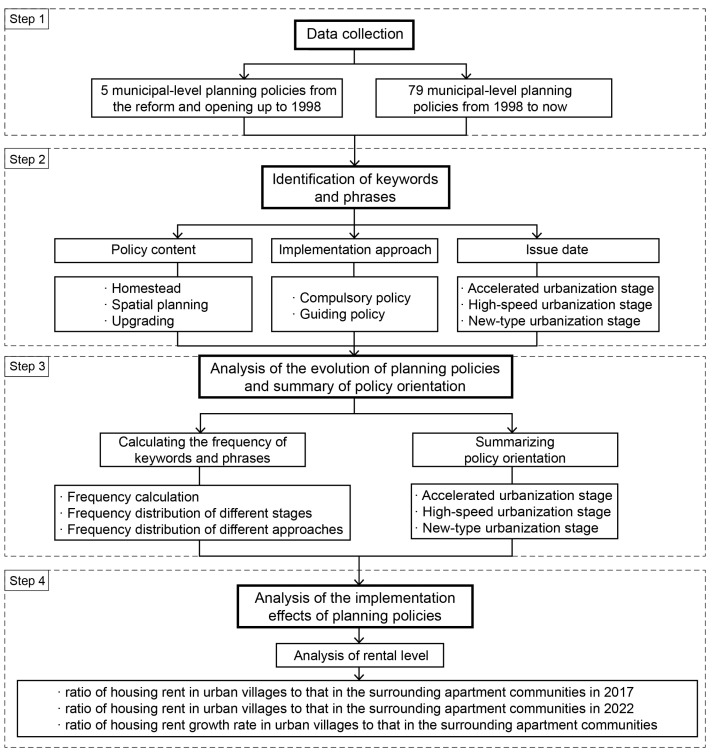
Methodological framework.

The methodological framework of the research is shown in Figure 4. Based on a stage-by-stage review of the urbanization process in Hangzhou according to the relevant policies, this research mainly analyzed the planning policies related to urban villages in Hangzhou from the reform and opening up to the present. The research data include 79 municipal-level policies related to urban village development in Hangzhou and were gathered from the “Gazette of the people’s government of Hangzhou municipality” (http://www.hangzhou.gov.cn/, accessed on 10 January 2022) from 1998 to the present. The data also include 5 related municipal-level policies mentioned in the relevant literature and the “Hangzhou Yearbook” from the reform and opening up to 1998. All 84 policies were recorded in a data set for keyword indexing. All the policies are given in “supplementary materials”.

This research then constructed a policy analysis framework based on textual content, implementation approach, and date of issue to analyze the evolution of the planning policies for urban villages in Hangzhou through the content analysis method. The textural content is divided into three categories: homestead, spatial planning, and upgrading. These are the main topics concerning policy responses to the informality of urban villages resulting from China’s urban–rural dual system during the rapid urbanization process. Moreover, the three inter-related topics are, respectively, the major content for each of the three main planning phases for urban villages, namely the early phase for land allocation and land use planning, the intermediate phase for spatial planning and construction, and the later phase for renovation. With the related policies pertaining to these topics throughout the total planning process of urban villages, the overall policy orientations of different urbanization stages would be illustrated. Therefore, these three dimensions may constitute a suitable policy analytical framework for this research. The content analysis method focuses on assessing how often a word or a phrase is used in the different texts and examining that content longitudinally to understand how events, knowledge, or perceptions evolve over time, aiming at rendering a precise and accurate account of the text [65]. Keywords or phrases were identified along the three dimensions of homestead, spatial planning, and upgrading according to the literature review. The frequency of each keyword or phrase was then calculated considering the date the policy was issued and the implementation approaches used. The first time a keyword or phrase appeared in a policy was recorded; however, the recording did not occur if the keyword or phrase appeared multiple times in the same policy [66]. The calculation of keywords frequency is shown in “supplementary materials”. This research also summarized the policy orientations of the different urbanization stages in Hangzhou.

To analyze the implementation effect of the planning policies, this article compared the housing rent of urban villages developed under different planning policy orientations with the housing rent of the surrounding apartment communities. We chose “rental level comparisons” to examine the implementation effects of planning policies for the following reasons. First, the housing rent level indicates the socioeconomic growth of urban villages, as well as how urban villages function for the two main groups of people, namely migrants and local villagers. According to the related literature, urban villages mainly function as appropriate accommodations for migrants and serve as the primary income source for local villagers [4,67]. Growth in the rental level may indicate a better rental market in the urban village and a higher degree of attraction for migrants, as well as increased income for local villagers. Additionally, other people-oriented evaluation indicators of human settlements and culture, such as living environment improvement, as well as preservation of the unique rural culture and tourism attraction enhancement may also improve the housing rent level of urban villages [68]. Therefore, rental level is an important factor in examining the development of urban villages for this research. Second, a comparison between the rental level of urban villages and their surrounding apartments indicates the difference in socioeconomic development between the urban villages and their surrounding urban environments, as well as the role of urban villages in the urban areas during the urbanization process. By comparing the indicators of the “rental level comparisons”, some interference factors, such as terrain, location, and surrounding urbanization level, could to some extent be avoided, since the urban villages and their surrounding apartment communities share a similar geographical environment. Moreover, the indicators of “rental level comparisons” may also help remove other interference factors, such as the spillover effect derived from the development of the surrounding urban built-up areas, since it influences the rental level of urban villages and their surrounding apartment communities in a similar way. Housing rent data were collected from the “National House Price Market Platform” (https://www.creprice.cn/, accessed on 22 May 2022), which provides data on the housing rent of urban villages and the surrounding apartment communities for the past five years. This paper is based on the collection of housing rent data in April 2017 and April 2022, and the calculation of the ratio of housing rent in urban villages to that in the surrounding apartment communities in April 2017 (X_2017_) and April 2022 (X_2022_), as well as the ratio of the housing rent growth rate in urban villages to that in the surrounding apartment communities (G), to compare the socioeconomic development of the urban villages under different planning policies. The mathematical equations utilized are shown in Equations (1), (2), and (3):

X_2017_ = R_2017_/r_2017_, (1)

X_2022_ = R_2022_/r_2022_, (2)

G = [(R_2022_/R_2017_)^(1/5)^ − 1]/[(r_2022_/r_2017_)^(1/5)^ − 1] (3)

R_2017_ is the housing rent in urban villages in April 2017, and r_2017_ is the rent in the surrounding apartment communities in April 2017. R_2022_ is the housing rent in urban villages in April 2022, and r^2022^ is the rent in the surrounding apartment communities in April 2022.

## 4. Policy Review and Implementation Effects Analysis

### 4.1. Review of the Urbanization Stages in Hangzhou

**Figure 5:**
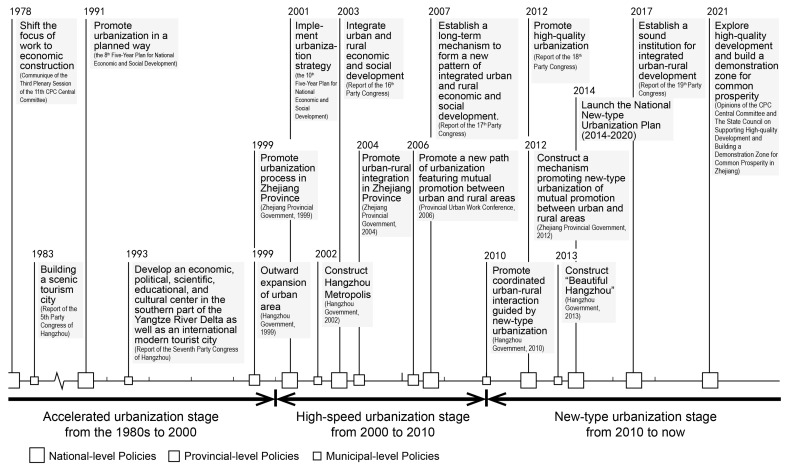
A stage-by-stage review of Hangzhou’s urbanization process.

According to the national, provincial, and municipal-level policies related to urbanization, this research divided the urbanization process in Hangzhou into three stages, namely, the accelerated urbanization stage from the reform and opening up to 2000, the high-speed urbanization stage from 2000 to 2010, and the new-type urbanization stage from 2010 to the present (Figure 5).

Since the reform and opening up, especially since the 1990s, China has begun to accelerate the process of urbanization. The 3rd Plenary Session of the 11th Central Committee of the CPC in 1978 stated the aim to shift the focus of the Party’s work to economic development. In 1984, the Central Committee of the CPC declared “the reform of the economic system with emphasis on cities”, emphasizing the reform and growth of the urban economy. In 1991, the concept of urbanization was first proposed in the Five-Year Plan for National Economic and Social Development [12], initiating a new stage of modernization in China. Since 1994, institutional adjustments, including reforms in the tax distribution system, the introduction of the land banking system, and reforms in the urban housing system, have accelerated urbanization, resulting in urban construction centered on land development [69,70]. Based on the national policy orientation, as well as its characteristic urban landscapes and cultural history, Hangzhou set a strategic goal in 1993 to develop an economic, political, scientific, educational, and cultural center in the southern part of the Yangtze River Delta, as well as an international modern tourist city, beginning to explore the development strategies for accelerated urbanization.

The high-speed urbanization stage in Hangzhou lasted from 2000 to 2010. In December 1999, the Zhejiang Provincial Government issued the first regional urbanization development plan in China, entitled “The Outline of Urbanization in Zhejiang Province”. This text signaled the beginning of a new stage of rapid urbanization in Zhejiang Province. Hangzhou, the capital city of Zhejiang Province, then started to implement a new development strategy advocating for high-speed urbanization and developing a networked metropolis [71]. Moreover, Hangzhou continuously implemented rural renovation projects since 2003, following the spirit of relevant provincial policies, aiming at improving the living conditions of villagers by supporting infrastructure construction and environmental renovation in Hangzhou’s rural areas.

Since 2010, Hangzhou has entered a new stage, intensively promoting new-type urbanization. In 2010, the Hangzhou government issued a policy titled “Implementation Opinions on further Strengthening Integrated Development in Urban and Rural Areas Mainly Through New-type Urbanization”, putting forward the goals of narrowing urban-rural gaps and improving the level and quality of urbanization. Moreover, two policies called “Outline for Constructing a Beautiful Hangzhou” and “Implementation Measures for Constructing Hangzhou-style Dwellings as Display” were released in 2013 and 2014, and they emphasized the requirements for the protection of natural landscapes and cultural heritage, highlighting the significance of rural resources in the process of urbanization.

In summary, Hangzhou has experienced the stages of accelerated urbanization and high-speed urbanization, which are considered traditional urbanization in this research, as well as new-type urbanization. Moreover, the development concept of urbanization has grown from focusing on urban construction to coordinating the development of urban and rural areas, and from enhancing the physical environment to achieving higher quality urbanization development that comprehensively exploits the natural and cultural value of villages. Following the policy orientation toward urbanization, Hangzhou has continuously adjusted its planning policies for urban villages.

### 4.2. Content Analysis of Planning Policies for Urban Villages

**Figure 6:**
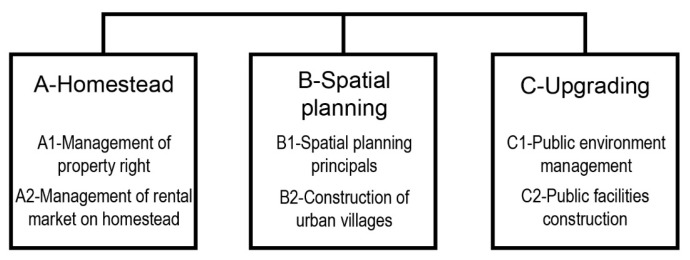
Three dimensions and six sub-classifications for policy analysis.

Following a stage-by-stage review of the urbanization process in Hangzhou, this article analyzed the 84 municipal-level planning policies related to urban villages in Hangzhou at different urbanization stages and identified 20 keywords/phrases through 246 indexing records based on the categories of homestead, spatial planning, and upgrading, covering six sub-classifications (Figure 6).

**Figure 7:**
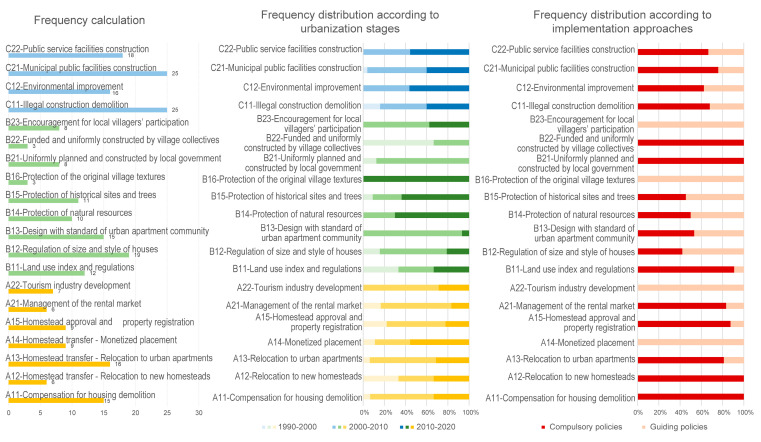
Frequency calculation of keywords and phrases.

This article calculates the frequency of each keyword/phrase and its frequency distribution according to the urbanization stages and implementation approaches. The results are shown in Figure 7.

The results indicate the following:

(1) For homestead transfers, Hangzhou has explored several strategies. Among them, the frequency of “A13-Relocation to urban apartments”, which refers to the replacement of homesteads with sets of urban apartments with formal property rights, is the highest, and the corresponding keywords are mainly found in policies from the high-speed urbanization stage to the present. Additionally, “A12-Relocation to new homesteads”, or relocating landless villagers to concentrated homesteads designated by the local government, appears less frequently. Compared with “A13-Relocation to urban apartments”, there is a higher proportion of “A12-Relocation to new homesteads” in the policies of the accelerated urbanization stage. Concerning the management of the rental market on the homestead, apart from allowing for an informal housing rental market with supervision (“A21-Management of the rental market”), the local government has also encouraged the development of the tourism industry, such as *nongjiale* and B&B (“A22-Tourism industry development”), since the high-speed urbanization stage to improve local residents’ income and the land value in urban villages based on their natural and cultural resources.
(2) In terms of spatial planning, policies related to “B11-Land use index and regulations” and “B12-Regulation on size and style of houses” have frequently appeared since the reform and opening up, acting as important rules for planning practices of urban villages. Additionally, the frequency of “B13-Design with the standard of urban apartment community” is relatively high, and the related policies are mostly found in the high-speed urbanization stage, indicating that Hangzhou has actively explored planning approaches replacing urban villages with urban apartments since the high-speed urbanization stage. Moreover, since the high-speed urbanization stage, policies related to “B14-Protection of natural resources” and “B15-Protection of historical sites and trees” have become more prevalent, suggesting that the Hangzhou government has placed a high priority on the protection of natural and cultural resources in urban villages. “B16-Protection of the original village textures” is mainly found in guiding policies during the period of new-type urbanization. It encourages the preservation of local villagers’ original homesteads and houses as well as street layouts to preserve urban villages’ unique textures formed by local villagers’ long-term, spontaneous, and informal planning practices to better exert the positive potential of informality in cultural inheritance. Regarding the construction of urban villages, “B22-Funded and uniformly constructed by the village committee”, according to which village committees are primarily in charge of the whole process of urban village construction, usually appears in the accelerated urbanization stage. “B21-Uniformly planned and constructed by local government” mostly appears in the high-speed urbanization stage, when the district governments were totally responsible for the construction of urban villages.
(3) In terms of upgrading, the frequencies of “C11-Illegal construction demolition” and “C21-Municipal public facilities construction” are the highest. The policies related to “C11-Illegal construction demolition” are relatively evenly distributed across the three stages of urbanization, acting as a key point in the planning practices for urban villages across the three urbanization stages. Policies related to “C12-Environmental improvement”, “C21-Municipal public facilities construction”, and “C22-Public service facilities construction” are mainly found from the high-speed urbanization stage to the present, indicating that the Hangzhou government has started to pay attention to the improvement in living environments, infrastructures, and public services in urban villages since the high-speed urbanization stage. Moreover, in the categories of “C12-Environmental improvement” and “C22-Public service facilities construction”, there is a higher proportion of guiding policies, aiming to stimulate the initiative of local villagers and village collectives through strategies such as “incentives instead of allocations” (yi-jiang-dai-bo) and “clarifying the responsibilities of local villagers for the public environments surrounding their houses” (men-qian-san-bao) to achieve long-term management of the public environment and rational allocation of the public service facilities in urban villages.
(4) Based on the policy content concerning homesteads, spatial planning, and upgrading, three policy orientations of Hangzhou’s different urbanization stages are summarized, namely limiting informality, preventing informality, and allowing some degree of informality (Table 1). The policy orientation of limiting informality is mostly developed in the accelerated urbanization stage, in which “relocation to new homesteads” is implemented, and the homestead area standard is set by each municipal district government. For example, in Xihu District in Hangzhou, the homestead area standard was set in “Regulations on the Administration of Private House Building in Rural Areas of Xihu District, Hangzhou (for trial implementation)” in 1999: the homestead of large households (more than six persons) should be no more than 125 m^2^; that of medium households (four to five persons) should be no more than 100 m^2^; and that of small households (fewer than three persons) should be no more than 75 m^2^. During this period, the village collective was in charge of carrying out unified planning and foundation construction in the new rural settlements designated by the local government, and the local villagers themselves raised the funds to construct the new houses according to the unified guidelines. These policies, to some extent, helped regulate local villagers’ practices during the housing construction process to limit the informality caused by their spontaneous and informal planning practices. The policy orientation of preventing informality is mostly developed in the high-speed urbanization stage. As a response to the issues of disorganized layout and difficulty in governance in the urban villages built by the village committee during the accelerated urbanization stage [72], the policies in the high-speed urbanization stage declare the district governments to be responsible for the uniform planning and construction of urban apartment communities for the concentrated relocation of landless villagers from multiple urban villages, without the involvement of village collectives and local villagers. According to the related policies, each household may receive two to three sets of apartments with a standard of 50 m^2^ per capita [72]. The district governments are responsible for the construction of the apartments and public facilities within the communities, with reference to the “Code for the planning and design of urban residential areas”. By replacing local villagers’ homesteads with the property rights of multi-story apartments constructed by the local government, villagers are no longer involved in the housing construction process, so the spontaneous and informal practices and the potential phenomenon of informality are prevented. The policy orientation of allowing some degree of informality is mostly developed in the new-type urbanization stage with fewer related policies. These policies are mainly applied to historical and cultural villages, traditional villages, villages in scenic spots, etc., with the main content including the objective to preserve the local villagers’ original homesteads and houses, as well as the distinctive textures of urban villages, which are created by long-term, spontaneous, and informal planning practices by local villagers, to encourage the villagers’ involvement in the redevelopment, reconstruction, and renovation of urban villages, to invest in infrastructure and public service upgrades developing tourism and the rental market, and to activate the development potential of urban villages.

### 4.3. Comparison of the Policy Implementation Effects

#### 4.3.1. Selection of Urban Villages for Comparison

In this research, urban villages in the Xihu District of Hangzhou were selected to examine the implementation effects of the planning policies of the different urbanization stages. Located in the western part of Hangzhou, Xihu District exhibits diverse landscapes with both plain and hilly terrains where there is a rich sample of urban villages developed under different planning policies. Additionally, Xihu District is located inside Hangzhou’s central urban area and has been exploring the planning policies for urban villages since the accelerated urbanization stage, making it suitable for analyzing the development of urban villages under the planning policies of the three urbanization stages. It should be noted that the planning policies for urban villages in Hangzhou are primarily implemented by the district governments. To prevent the disturbance of differences in the implementation across district governments in the results, this paper focuses on urban villages within the same administrative district (Xihu District) to better compare the implementation effects of different planning policies.

**Figure 8:**
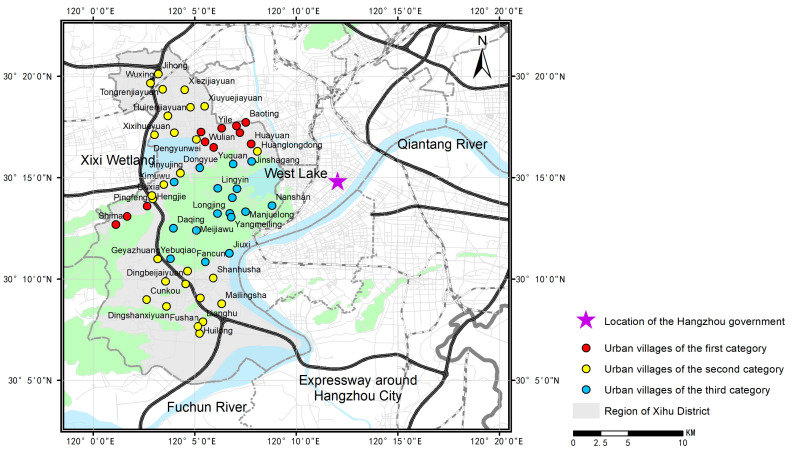
Spatial distribution of the selected urban villages for comparison.

Based on the content analysis of planning policies for urban villages in Hangzhou, this article categorizes the urban villages in Xihu District into three types: the first category—urban villages developed through planning policies that limit informality; the second category—urban villages developed through planning policies that prevent informality; and the third category—urban villages developed through planning policies that allow some degree of informality. All the urban villages of different categories are listed in Table 2, and the spatial distribution of the urban villages is shown in Figure 8. The majority of urban villages of the first and second categories are found in the plain area, while those of the third category are found in the hilly area, indicating that the planning policies developed in the accelerated and high-speed urbanization stages were mostly applied to the urban villages in the plain areas, while the planning policies developed in the new-type urbanization stage were mostly applied to the urban villages in the hilly areas. It should be noted that the urban villages of the third category are not new urban villages formed after 2010, but urban villages that, due to geographical constraints, were not included in the formal urban development plan and policies during the traditional urbanization stage. These urban villages were thus developed spontaneously and informally before being developed under the planning policies guided by new-type urbanization that allow some degree of the informality created before. Therefore, the urban villages of the third category have undergone the traditional and new-type urbanization stage and may be comparable with the urban villages of the first and second category in examining the long-term implementation effect of planning policies and strategies guided by traditional and new-type urbanization. Moreover, the proportion of urban villages of the second category is the highest, demonstrating that the main planning policy orientation for urban villages in Hangzhou continues to be preventing informality, which is developed in the high-speed urbanization stage.

#### 4.3.2. Comparison of Policy Implementation Effects

This article calculated the ratio of housing rent in urban villages to that in the surrounding apartment communities in April 2017 (X_2017_) and April 2022 (X_2022_), as well as the ratio of the housing rent growth rate in urban villages to that in the surrounding apartment communities (G). One-way analysis of variance (ANOVA) for the three sets of data was performed. After excluding the demolished urban villages, urban villages lacking suitable surrounding apartment communities for comparison, and urban villages that have not been rebuilt or rented yet, this article analyzes the differences in the rent level of the remaining urban villages, and the results are shown in Table 3. According to the results, there is no significant difference in X_2017_ among the three categories of urban villages. However, there is a significant difference in X_2022_ among the three categories of urban villages, indicating that the difference in rental level ratio among the three categories of urban villages may be increasingly widening. The mean of X_2022_ for urban villages of the third category is higher than 1 and is significantly higher than that of the other two categories of urban villages, while there is no difference in X_2022_ between the urban villages of the first and second category, indicating that allowing some degree of informality may make it easier to realize a higher rental level in the urban villages than in the surrounding apartment communities. Additionally, the growth rate in housing rent in the urban villages of the third category is higher than that in their surrounding communities, while that in the urban villages of the first and second category is lower, indicating that the urban villages under the policies allowing some degree of informality may have greater development potential, while the development of the other two categories of urban villages may be limited. It is suggested that compared with the planning policies limiting and preventing informality, the planning policies allowing some degree of informality may be more conducive to realizing the long-term socioeconomic development of urban villages.

**Figure 9:**
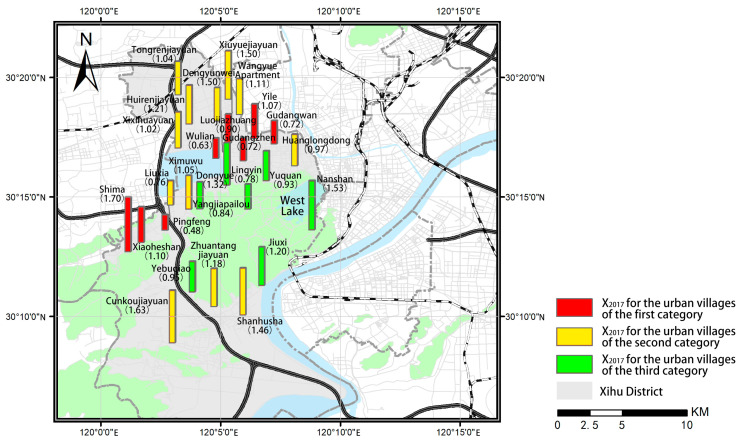
Spatial distribution of X_2017_ for the urban villages of the three categories.

**Figure 10:**
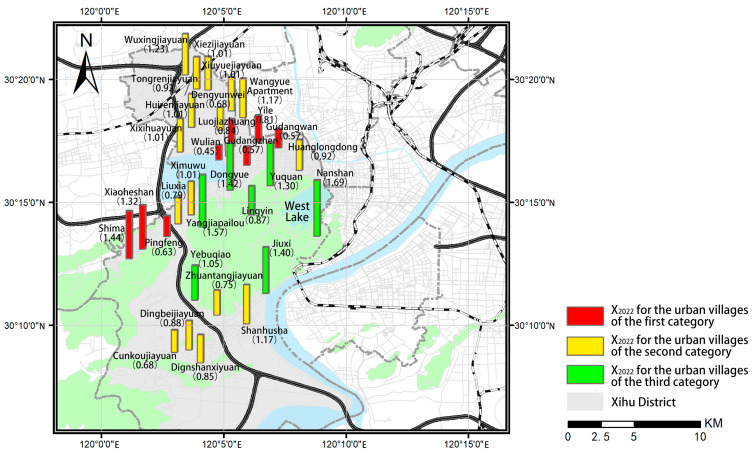
Spatial distribution of X_2022_ for the urban villages of the three categories.

**Figure 11:**
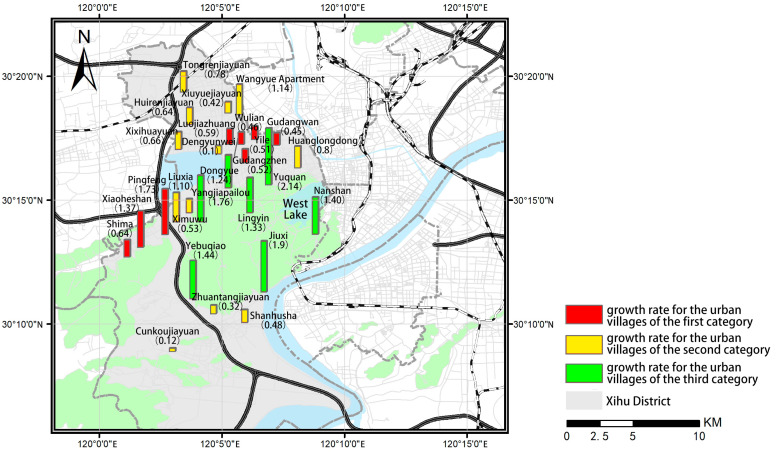
Spatial distribution of the growth rate for the urban villages of the three categories.

The results also indicate a certain degree of difference among the urban villages within the same category. We further perform the spatial distribution analysis of X_2017_, X_2022_, and G for the urban villages of the three categories (Figure 9, Figure 10 and Figure 11) and examine the actual policy implementation to explore the potential influencing factors.

**Figure 12:**
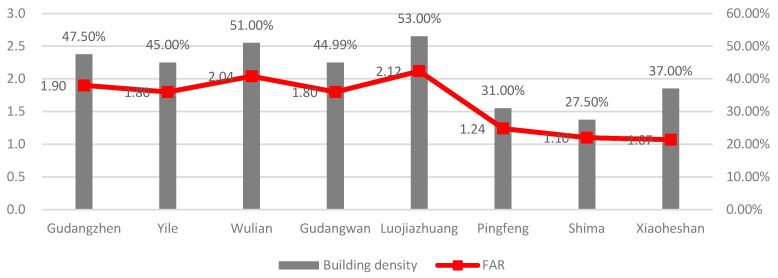
Building density and FAR of the urban villages of the first category.

The most notable difference is found in the urban villages of the first category. Pingfeng, Xiaoheshan, and Shima stand out among them as exhibiting a noticeably higher rental level ratio to their surrounding apartment communities than other urban villages of the first category do. From the perspective of the homestead, Pingfeng, Shima, and Xiaoheshan, which were relocated later, received a slightly lower standard of homestead allocation compared with Luojiazhuang, Wulian, and other urban villages, which were relocated earlier. Luojiazhuang and Wulian, for example, completed the relocation in the early 1990s, and their homestead allocation standards were 110 m^2^ for large households (six persons and more) and 100 m^2^ for medium households (four to five persons). Pingfeng, Shima, and Xiaoheshan began the relocation process from 2002 to 2004, and the new homestead allocation standards were 100–110 m^2^ for large households, 88 m^2^ for medium households, and about 60 m^2^ for small households (three or fewer than three persons) (Table 4). The relatively small homestead area standard may provide greater room for public places and green spaces in the new settlements. In terms of spatial planning, the village collectives of the three urban villages commissioned qualified design institutes for the unified design and construction of the new settlements. They partially learned from the community planning and design experiences of the other urban villages of the same category built earlier and avoided local villagers’ illegal construction to a greater extent. Moreover, compared to the urban villages found in the core urban area, the three urban villages are located in the foothills, where there is still a small amount of farmland, and the requirement for land development intensity is relatively lower. Therefore, the density and FAR of the three urban villages are significantly lower than the average level of those in other urban villages (Figure 12); more public spaces and green spaces could be placed during both the construction and upgrading process, providing better living environments compared with other urban villages of the first category. It is indicated that creating and optimizing public spaces, which greatly improve the built environment of urban villages, may be beneficial for the long-term socioeconomic development of the urban villages of the first category.

Additionally, there is also an evident difference in the rental level ratio to the surrounding apartment communities among the urban villages of the third category. Among them, Nanshan exhibits a significantly higher ratio in rental level, and Yuquan and Jiuxi exhibit a significantly higher ratio in growth rate. Compared to other urban villages of the third category, Nanshan, Jiuxi, and Yuquan exhibit a significant difference in the upgrading process. These three urban villages are all located at the junction between the scenic spot and the urban area and are closest to the West Lake, closest to the urban CBD, and closest to the urban main road among the urban villages of the third category, respectively, exhibiting significant geographical advantages. Relying on the relatively mature urban life functions of the surrounding area, Nanshan introduced cultural and creative industries, including internet celebrity restaurants, boutique homestays, photography studios, exhibition halls, personalized brand clothing, and accessories in the upgrading project in 2015, realizing the transformation and development of the urban village. Taking advantages of its ideal location, natural environment, and characteristic rural landscapes, Yuquan Village spontaneously attracts a large number of catering businesses and popularity. In 2016, a large number of individualized B&Bs were introduced in the upgrading project, and the urban villages were transformed into an attraction for tourism and a famous slow-life leisure block in Hangzhou. At present, there are 238 businesses in Yuquan Village, including 115 themed B&Bs, 82 specialty restaurants, and 21 other businesses. In 2019, the business revenue reached CNY 175 million, and the tourist flow exceeded CNY 1.7 million. Jiuxi Village has also attracted a large number of tourists due to its geographical advantages, natural landscape, and cultural resources and has gradually become one of the most famous places for outdoor camping, home parties, and barbecue in Hangzhou. Therefore, based on a good location and unique rural cultures, introducing new industries, such as cultural and creative industries, to enhance tourism attraction would be advantageous for the socioeconomic transformation and development of the urban villages of the third category.

Regarding the urban villages of the second category, the difference in the rental level ratio is relatively smaller than that of the other two categories. Among the urban villages of the second category, Liuxia and Wangyue Apartments exhibit a significantly higher ratio to their surrounding apartment communities in the growth rate of rental levels. Compared to other urban villages, Wangyue is a typical urban village near the campus and has a higher proportion of students. After construction, local villagers were allowed to flexibly transform the houses into several single rooms measuring 15–50 m^2^ according to the diversified living needs of students and other tenants. This may have increased its attraction to students and the surrounding tenants, as well as the housing rent. Liuxia is a resettlement apartment community built in 2000. Before the implementation of the upgrading project in 2021, there were problems with backward infrastructure, outdated parking facilities, and relatively low rent. After collecting suggestions from local residents and organizing several residents’ representatives to discuss and formulate the upgrading plan, the community began to implement 30 renovation items, including the demolition of illegal buildings, the addition of centralized activity places, the addition of leisure facilities, and the installation of elevators, which significantly improved the living environment of the community and may be more conducive to the development of housing rent in urban villages. In conclusion, flexible house design and environmental improvement according to the real needs of local residents may be conducive to the improvement of rent levels and long-term socioeconomic development of the urban villages of the second category. In summary, compared with the planning policies limiting and preventing informality, the planning policies allowing some degree of informality may be more conducive to realizing the long-term socioeconomic development of urban villages. Moreover, differentiated strategies could be implemented in different categories of urban villages to better activate their development potentials.

## 5. Discussion

According to the results, informality in urban villages may have positive power, which cannot be replaced by formality and should be given more attention in policy making during the urbanization process. This section goes into further detail on how the planning policies guided by new-type urbanization make use of informality and improve the socioeconomic development of urban villages.

**Figure 13:**
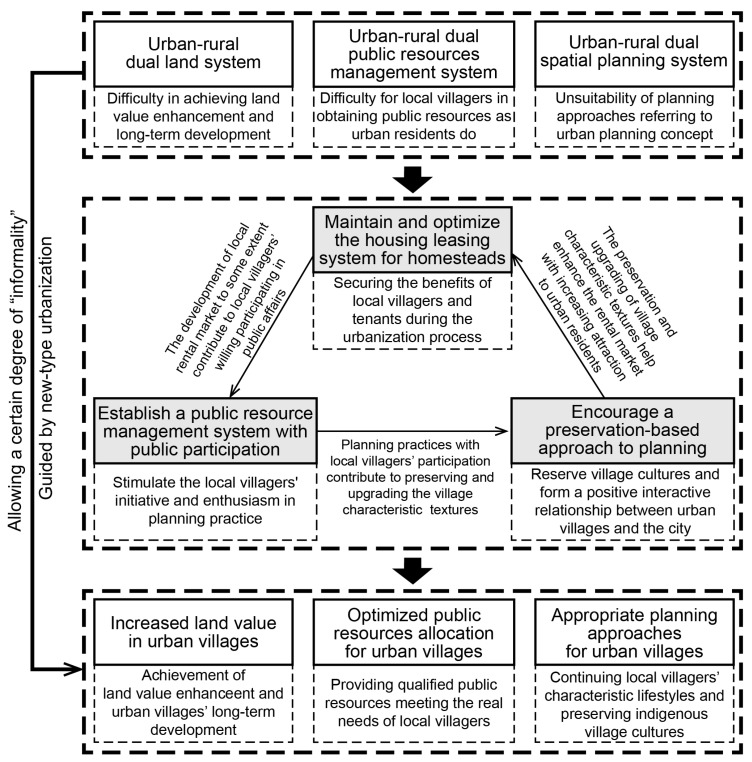
Diagram of planning policies and practices allowing some degree of informality guided by new-type urbanization.

Guided by new-type urbanization, the Hangzhou government has explored policies seeking to maintain and optimize the local housing leasing system for homesteads, establish a public resource management system with public participation, as well as encourage a character-preservation-based approach for spatial planning, which may make better use and exert the positive power of the informality in urban villages and overcome the institutional barriers of urban-rural dual structures incrementally (Figure 13). First, maintaining and optimizing the local housing leasing system for homesteads contributes to securing the benefits of local villagers and tenants during the urbanization process. By preserving and improving the informal housing and business, the existing property rights and land market that spontaneously formed based on the local conditions are protected to a certain extent, thus minimizing the potential harm to the interests of local villagers and tenants and better safeguarding people’s “real property rights” and long-term development rights [73,74]. Second, establishing a public resource management system with public participation helps stimulate local villagers’ initiative and enthusiasm for planning practices for urban villages. The government’s intervention in enhancing the public environment and allocating public resources, as well as encouraging local villagers to participate in planning practices for urban villages, can, to a certain extent, improve the infrastructure and complement the existing top-down control-based planning practices for urban villages [75,76], achieving more rational public resource allocation and planning practices that are more flexible and adapted to local realities [77]. Third, encouraging a preservation-based approach to spatial planning contributes to preserving the characteristic natural and cultural resources of urban villages and fostering a positive interactive relationship between urban villages and the city. The characteristic village fabric formed by local villagers’ long-term, spontaneous, and informal practices carries the daily social activities in urban villages, displaying a living culture with authenticity and dynamic development [68] and serving as an important part of the natural and cultural resources of the city, which may also enhance the land value and development potential of urban villages under the local government’s guidance in industrial transformation and contribute to the overall development during the urbanization process.

Therefore, guided by new-type urbanization, which emphasizes the people’s long-term development right with the concept of urban villages’ active participation in the urbanization process and dynamic complementarity and symbiosis with cities, allowing a certain degree of informality contributes to breaking through the institutional barriers of the urban-rural dual structure, including land systems, public resource allocation systems, and spatial planning systems, and realizing the upshifting of land value, rational allocation of public resources, and characteristic planning approaches for urban villages better suited to the long-term development of urban villages during the urbanization process.

It should be noted that the findings and policy implications of this research are context specific and need site-specific analysis before being replicated in other countries or regions. First, Chinese urban villages exhibit uniqueness compared to other informal settlements characterized as illegal or temporary invasions and shortage of services provision in Latin America, southeast Europe, and some countries in Asia [78,79,80]. Chinese urban villages distinguish themselves at least with their rural social life, stable kinship ties, and characteristic village textures, which are conceived as a form of informality and derived from the villages’ long cultural histories, believed to be an engine for further development of urbanization. Second, in the Chinese setting, where formal planning predominates in the Chinese planning system, the positive power of informality for urban village development has also received less research. By taking Hangzhou, a pioneer city in practicing new-type urbanization combining the characteristics of rapid urbanization, a characteristic geographical environment, and a long historical culture, as a typical case study, this research benefited from the opportunity to observe and compare different planning policies and attitudes responding to the informality of urban villages in the same space–time context and concluded with new attitudes and policy implications concerning informality in Chinese urban villages. Since the discoveries are derived from a specific case study, more related research in other cultural contexts should be conducted to further explore and prove the positive role of informality in the urbanization process.

## 6. Conclusions

This article focuses on the planning policies of urban villages in Hangzhou, China, aiming to compare the influences of planning policies for urban villages guided by traditional and new-type urbanization on urban village development. This research establishes a multi-dimensional policy analysis framework based on policy contents, implementation methods, and issue dates to analyze the evolution of the municipal-level planning policies for urban villages in Hangzhou, China. The article further compares the rental level, a primary indicator describing socioeconomic development, of urban villages in Xihu District in Hangzhou developed under the planning policies of different urbanization stages to analyze their implementation effects. The results indicate that guided by new-type urbanization, the policy orientation for urban villages in Hangzhou has shifted from limiting and preventing informality to allowing a certain degree of informality, realizing a higher rental level for urban villages. It is suggested that the informality of urban villages may have positive power, which cannot be replaced by formality and deserves greater consideration in the policy making during the urbanization process to achieve urban villages’ co-prosperity with cities.

Compared with the previous literature related to urban villages, this study analyzes the development of urban villages from the perspective of new-type urbanization, opening up a new direction for the study of urban village development and providing implications for the related studies on urban and rural settlements during the rapid urbanization process. Moreover, this research discusses new attitudes and responses toward informality during the urbanization process—an important issue for the rapidly urbanizing regions of the global south and some regions of the global north experiencing urban transition—and establishes a policy analysis framework based on the urban-rural dual structure, serving as a reference for policy analysis regarding urban–rural interaction during the rapid urbanization process for these regions. Furthermore, the findings of this research advocate the view that informality is not only a survival strategy for middle- or low-income people in developing countries, but it is also profoundly related to the local cultural inheritance [68]. This research further highlights the positive power of informality, which formality cannot replace, and provides policy implications toward informality during the urbanization process that may be replicated in other regions with similar contexts and circumstances, enriching the formality–informality debates and application in developing countries. In future research, case studies may be adopted to observe the long-term development of urban villages under the different planning policies and analyze the interactions between local governments’ uniform and formal planning and local residents’ spontaneous and informal planning practices to provide more detailed policy guidance for urban villages.

## Figures and Tables

**Table 1:** Summary of the three policy orientations of the different urbanization stages.

Categories of Policies	Policy Content	Policy Orientations		
		Limiting Informality	Preventing Informality	Allowing Some Degree of Informality
Homestead	Relocation to new homesteads	○		
	Relocation to urban apartments		○	
	Management of housing rental market for migrants	○	○	○
	Tourism industry development			○
Spatial planning	Land use index and regulations	○	○	○
	Regulations on size and style of houses	○	○	○
	Design with standard of urban apartment community		○	
	Protection of the original village textures			○
	Uniform planning and construction by local government		○	
	Funding and uniform construction by village collectives	○		
	Encouragement of local villagers’ participation in construction			○
Upgrading	Illegal construction demolition/Environmental improvement/Municipal public facilities construction	○	○	○
	Public service facilities construction		○	○

○ Dominated by compulsory policies; ○ Dominated by guiding policies.

**Table 2:** List of the urban villages developed through the three policy orientations.

Type of Urban Villages	List of Urban Villages
Category 1—Urban villages developed through planning policies that limit informality	Gudangzhen, Yile, Wulian, Gudangwan, Baoting ^1^, Songjiang ^1^, Huayuan ^1^, Luojiazhuang, Pingfeng, Shima, Xiaoheshan
Category 2—Urban villages developed through planning policies that prevent informality	Huanglongdong, Dengyunwei, Xixihuayuan, Jiangcuhuayuan ^2^, Zhuantangjiayuan, Zhijiangjiayuan ^2^, Cunkou, Dingbeijiayuan, Dingshanxiyuan, Shanhusha, Huilong ^2^, Lianghu ^2^, Fushan ^3^, Geyazhaung ^3^, Mailingsha ^3^, Wuxingjiayuan, Xiezijiayuan, Jihong ^2^, Wangyue Apartment, Tongrenjiayuan, Xiuyuejiayuan, Huirenjiayuan, Liuxia, Hengjie^3^, Ximuwu, Jinyujing ^3^
Category 3—Urban villages developed through planning policies that allow some degree of informality	Yuquan, Nanshan, Shuangfeng ^2^, Jiuxi, Longjing ^2^, Yangmeiling ^2^, Maojiabu ^2^, Fancun ^2^, Meijiawu ^2^, Lingyin, Wengjiashan ^2^, Jinshagang ^2^, Manjuelong ^2^, Dongyue, Yangjiapailou, Daqing ^2^, Yebuqiao

^1^ urban villages of the first category that have been demolished; ^2^ urban villages with no suitable surrounding apartment communities for comparison of rental levels; ^3^ urban villages that have not been rebuilt or rented yet.

**Table 3:** Comparison of the housing rent in the three types of urban villages through ANOVA.

		Average	Standard Deviation	*p* Value	F Value	Multiple Comparisons after ANOVA		
						Categories	Variations in Difference	*p* Value
X_2017_	Type one	0.95	0.37	0.421	0.898	Category 1	−0.17	0.197
						Category 2	−0.13	0.394
	Type two	1.12	0.20			Category 1	0.17	0.197
						Category 3	0.44	0.745
	Type three	1.08	0.28			Category 1	0.13	0.394
						Category 1	−0.44	0.745
X_2022_	Type one	0.85	0.35	0.010 **	5.484	Category 1	−0.09	0.392
						Category 2	−0.41	0.004 **
	Type two	0.94	0.17			Category 1	0.09	0.392
						Category 3	−0.31	0.010 *
	Type three	1.26	0.28			Category 1	0.41	0.004 **
						Category 1	0.31	0.010 *
G	Type one	0.78	0.49	0.000 **	16.143	Category 1	0.17	0.321
						Category 2	−0.82	0.000 **
	Type two	0.61	0.31			Category 1	−0.17	0.321
						Category 3	−1.00	0.000 **
	Type three	1.61	0.34			Category 1	0.82	0.000 **
						Category 1	1.00	0.000 **

* *p* < 0.05; ** *p* < 0.01.

**Table 4:** Standard of homestead allocation for urban villages of the first category.

Name of Urban Villages	Wulian, Luojiazhuang (Built in the Early 1990s)	Yile, Gudangzhen, Gudangwan (Built in the Late 1990s)	Xioheshan, Pingfeng, Shima (Built in the Early 2000s)
Standard of homestead allocation	110 m^2^ for households of six persons and more; 100 m^2^ for households of four to five persons	100 m^2^ for households of five persons and more; 80 m^2^ for households of three to four persons; 60 m^2^ for households of one to two persons	100–110 m^2^ for households of six persons and more; 88 m^2^ for households of four to five persons; 60 m^2^ for households of fewer than three persons

## Data Availability

The data presented in this study are available on request from the corresponding author.

## Supplementary Materials

The following supporting information can be downloaded at: https://www.mdpi.com/article/10.3390/ijerph192416596/s1.
